# 1,4-Dihydropyridine as a Promising Scaffold for Novel Antimicrobials Against *Helicobacter pylori*

**DOI:** 10.3389/fmicb.2022.874709

**Published:** 2022-05-25

**Authors:** Andrés González, Javier Casado, Miyase Gözde Gündüz, Brisa Santos, Adrián Velázquez-Campoy, Cristina Sarasa-Buisan, María F. Fillat, Milagrosa Montes, Elena Piazuelo, Ángel Lanas

**Affiliations:** ^1^Group of Translational Research in Digestive Diseases, Institute for Health Research Aragón (IIS Aragón), Zaragoza, Spain; ^2^Department of Medicine, Psychiatry and Dermatology, University of Zaragoza, Zaragoza, Spain; ^3^Institute for Biocomputation and Physics of Complex Systems (BIFI), Zaragoza, Spain; ^4^Biomedical Research Networking Center in Hepatic and Digestive Diseases (CIBERehd), Madrid, Spain; ^5^Department of Biochemistry and Molecular and Cellular Biology, University of Zaragoza, Zaragoza, Spain; ^6^Department of Pharmaceutical Chemistry, Faculty of Pharmacy, Hacettepe University, Ankara, Turkey; ^7^Fundación Agencia Aragonesa para la Investigación y el Desarrollo (ARAID), Zaragoza, Spain; ^8^Department of Microbiology, Donostia University Hospital-Biodonostia Health Research Institute, San Sebastian, Spain; ^9^Biomedical Research Networking Center in Respiratory Diseases (CIBERES), Madrid, Spain; ^10^Aragón Health Sciences Institute (IACS), Zaragoza, Spain; ^11^Digestive Diseases Service, University Clinic Hospital Lozano Blesa, Zaragoza, Spain

**Keywords:** *Helicobacter pylori*, HsrA, hexahydroquinoline, novel antimicrobial drugs, antibiotic resistance, dihydropyridine

## Abstract

The increasing occurrence of multidrug-resistant strains of the gastric carcinogenic bacterium *Helicobacter pylori* threatens the efficacy of current eradication therapies. In a previous work, we found that several 1,4-dihydropyridine (DHP)-based antihypertensive drugs exhibited strong bactericidal activities against *H. pylori* by targeting the essential response regulator HsrA. To further evaluate the potential of 1,4-DHP as a scaffold for novel antimicrobials against *H. pylori*, we determined the antibacterial effects of 12 novel DHP derivatives that have previously failed to effectively block L- and T-type calcium channels. Six of these molecules exhibited potent antimicrobial activities (MIC ≤ 8 mg/L) against three different antibiotic-resistant strains of *H. pylori*, while at least one compound resulted as effective as metronidazole. Such antimicrobial actions appeared to be specific against *Epsilonproteobacteria*, since no deleterious effects were appreciated on *Escherichia coli* and *Staphylococcus epidermidis*. The new bactericidal DHP derivatives targeted the *H. pylori* regulator HsrA and inhibited its DNA binding activity according to both *in vitro* and *in vivo* analyses. Molecular docking predicted a potential druggable binding pocket in HsrA, which could open the door to structure-based design of novel anti-*H. pylori* drugs.

## Introduction

Chronic infection of the human gastric mucosa with the microaerophilic Gram-negative bacterium *Helicobacter pylori* can cause a variety of upper gastrointestinal diseases, including chronic gastritis, peptic ulcer, gastric mucosa-associated lymphoid tissue (MALT) lymphoma, and gastric cancer (GC; Kusters et al., 2006). Notably, more than a half of the world’s population is estimated to be infected with this microbial type I carcinogen (World Health Organization, 1994; Hooi et al., 2017). Unless properly treated, about 1% of the *H. pylori* infected people is estimated to develop GC along their lives (Kuipers, 1999). This calculation means that more than 4 million persons worldwide are currently at high risk or actually suffering GC due to untreated or mistreated *H. pylori* infections.

Eradication of *H. pylori* infection significantly reduces the risk of GC (Leung et al., 2018; Nam et al., 2019), even in persons with a family history of this malignancy in first-degree relatives (Choi et al., 2020). However, the increasing occurrence of multidrug-resistant strains of this clinically relevant pathogen worldwide begins to limit the efficacy of current eradication therapies (Boyanova et al., 2016, 2019; Savoldi et al., 2018). In 2017, the WHO included *H. pylori* in its first ever list of antibiotic-resistant “priority pathogens,” a catalogue of 12 families of bacteria that pose at present the greatest threat to human health, for which novel classes of antibiotics are urgently needed (Tacconelli et al., 2018). As consequence, multiple efforts are being made to discover and develop new therapeutic options, including both drug repurposing and *de novo* identification of bioactive compounds directed against novel and validated therapeutic targets in *H. pylori* (Salillas et al., 2019; González et al., 2019a,b, 2020, 2021; Roszczenko-Jasinska et al., 2020; Salillas and Sancho, 2020).

The *H. pylori* OmpR-like “orphan” response regulator HsrA (also known as HP1043) constitutes a promising therapeutic target (González et al., 2019a,b). This protein is unique and highly conserved among *Epsilonproteobacteria* (Muller et al., 2007), and its expression appears essential for cell viability (Beier and Frank, 2000; McDaniel et al., 2001). HsrA modulates the transcription of a plethora of genes and operons involved in relevant physiological processes including translation, transcription, chemiotaxis, energy metabolism, nitrogen metabolism and redox homeostasis (Delany et al., 2002; Olekhnovich et al., 2014; Pelliciari et al., 2017). Hence, the protein acts as a global homeostatic regulator, synchronizing metabolic functions and virulence with the availability of nutrients and cell division. In a previous work, we found that several 1,4-dihydropyridine (DHP)-class antihypertensive and highly prescribed drugs, such as nifedipine, nicardipine, nisoldipine, nimodipine, nitrendipine, and lercanidipine acted as low-molecular-weight ligands of HsrA and noticeably inhibited its *in vitro* DNA binding activity (González et al., 2019a). Some of these HsrA inhibitors exhibited potent bactericidal actions against different strains of *H. pylori*, including both clarithromycin- and metronidazole-resistant strains, and showed additive interactions with first-line antibiotics in checkerboard assays. Experimental therapies with 100 mg/kg/day of marketed formulations of nimodipine or nitrendipine, in combination with omeprazole (140 mg/kg/day) daily during 7 days, led to significant reductions in the *H. pylori* (strain PMSS1) gastric colonization in mice (González et al., 2019a). These results strongly supported the use of 1,4-DHPs as novel repurposable antimicrobial drugs against *H. pylori*; however, the repositioning of these antihypertensive drugs as antimicrobials could be linked to undesirable side effects associated with their intrinsic vasodilatation action and therefore the occurrence of potential hypotensive effects in both hypertensive and non-hypertensive patients. In this context, the use of 1,4-DHP as a scaffold for novel derivatives with similar or enhanced antimicrobial actions and mitigated side effects appears as a promising strategy for the development of novel antibiotics.

A new class of DHP derivatives, in which substituted cyclohexane rings are fused to 1,4-DHP forming a hexahydroquinoline (HHQ) group, has been previously achieved *via* modified Hantzsch reactions and tested for their L- and T-type calcium channel blocking activities by whole-cell patch clamp technique (Schaller et al., 2018; Aygün Cevher et al., 2019). Notably, some of these DHP-based HHQ derivatives failed to effectively block L- and T-type calcium channels. In the present study, we determined the anti-*H. pylori* activities of 12 condensed DHPs with no significant effect on calcium channels, and analyzed how different substituents on the molecule backbone affected the antimicrobial activity. At least six of these DHP derivatives demonstrated potent bactericidal activities against three antibiotic-resistant strains of *H. pylori*. As previously observed with commercial 1,4-DHP class antihypertensive drugs, the new DHPs appeared to target the essential response regulator HsrA according to both *in vitro* and *in vivo* evidences. The results further support the use of 1,4-DHP as a promising scaffold for novel antimicrobial drugs against *H. pylori*.

## Materials and Methods

### Chemicals

DHP-based HHQ derivatives were synthesized by the reaction of dimethyl-1,3-cyclohexanedione, substituted benzaldehyde, appropriate alkyl acetoacetate, and ammonium acetate as previously described (Schaller et al., 2018; Aygün Cevher et al., 2019), and stored as neat solid compounds at −20°C in amber tubes until use. DHP-class antihypertensive drugs were purchased from Sigma-Aldrich (Saint Louis, MO, United States), and properly stored according to the manufacturer’s instructions. Stock solutions of each 1,4-DHP derivative were freshly prepared at 20 mM in 100% dimethyl sulfoxide (DMSO) for electrophoretic mobility shift assays and isothermal titration calorimetry analyses, and at 10.24 g/L in 100% DMSO for determination of minimal inhibitory/bactericidal concentrations. Since DHPs are light-sensitive compounds, all stock solutions were protected from light. Metronidazole, clarithromycin, levofloxacin, and ampicillin were obtained from Sigma-Aldrich. Stock solutions of these antibiotics in 100% DMSO were prepared at 10.24 g/L and stored at −20°C for up to 30 days.

### Bacterial Strains and Culture Conditions

*Helicobacter pylori* reference strains ATCC 43504 (metronidazole-resistant) and ATCC 700684 (clarithromycin-resistant) were purchased from the American Type Culture Collection (Rockville, MD, United States). The *H. pylori* clinical isolate Donostia 2, resistant to levofloxacin, was isolated from gastroduodenal biopsies at Donostia University Hospital (San Sebastian, Spain). The *H. pylori* strain 26695 (ATCC 700392) was used in some experiments. All *H. pylori* strains were routinely grown in Blood Agar Base No. 2 (OXOID, Basingstoke, United Kingdom) supplemented with 8% defibrinated horse blood (OXOID) in a humidified microaerobic incubator (85% N_2_, 10% CO_2_, and 5% O_2_) at 37°C for 48–72 h. For certain experiments, bacteria were grown for 48–72 h at 37°C in brain heart infusion broth (OXOID) supplemented with 4% fetal bovine serum (Gibco, Carlsbad, CA, United States).

*Escherichia coli* ATCC 25922 and *Staphylococcus epidermidis* ATCC 12228 were kindly provided by Jose Antonio Aínsa (University of Zaragoza), and belong to the microbial culture collection of the Department of Microbiology, from this university. Both strains were routinely cultured overnight in Mueller-Hilton agar/broth (PanReac AppliChem, Barcelona, Spain) at 37°C.

### Minimal Inhibitory and Bactericidal Concentrations

Minimal inhibitory concentrations (MICs) were determined by the microdilution method using sterile 96-well flat-bottom microtiter plates as previously described (González et al., 2020), with slight modifications. Briefly, inoculum suspensions of *H. pylori* strains ATCC 43504 (metronidazole-resistant), ATCC 700684 (clarithromycin-resistant), and Donostia 2 (levofloxacin-resistant) were freshly prepared from cultures grown during 48 h at 37°C on Blood Agar Base No. 2 supplemented with 8% defibrinated horse blood under microaerobic conditions (85% N_2_, 10% CO_2_, and 5% O_2_). Bacterial growth from two blood agar plates was aseptically resuspended in 10 ml of brain heart infusion (BHI) broth supplemented with 4% fetal bovine serum (BHI + FBS) and next diluted to OD_600_ = 0.01 [~10^6^ colony forming units (CFU) per ml] in the same medium. A range of concentrations from 64 to 0.031 mg/L was tested for each 1,4-DHP derivative against each *H. pylori* strain. DMSO (vehicle) and conventional antibiotics were included as controls in all assays. Microtiter plates were incubated under microaerobic conditions at 37°C for 48 h; then, inhibition of microbial growth was colorimetrically revealed after the addition of filter-sterilized resazurin (Sigma-Aldrich, Saint Louis, MO, United States) to a final concentration of 0.01 mg/ml, and further incubation of 6 h. To determine the minimal bactericidal concentration (MBC), 10 μl aliquots of several dilutions around the MIC value were aseptically seeded on blood agar plates and incubated at 37°C for 72 h under microaerobic conditions. Each experiment was performed twice in triplicate.

Antimicrobial activities of selected compounds against the *E. coli* reference strain ATCC 25922 and the *S. epidermidis* reference strain ATCC 12228 were determined according to the EUCAST Guidelines [European Committee for Antimicrobial Susceptibility Testing (EUCAST) of the European Society for Clinical Microbiology and Infectious Diseases (ESCMID), 2003]. Briefly, standardized inoculums equivalent to a 0.5 McFarland standard turbidity (~1.5 × 10^8^ CFU per ml) were freshly prepared from overnight colonies on Mueller–Hinton agar plates of both microorganisms. Final bacterial suspensions at 5 × 10^5^ CFU per ml in Mueller–Hinton broth were faced to a range of concentrations from 64 to 0.031 mg/L of selected DHP derivatives. DMSO and ampicillin were included as controls in all assays. Plates were incubated at 37°C overnight, and MIC values were colorimetrically defined by the addition of 0.01 mg/ml resazurin. Aliquots were seeded on Mueller–Hinton agar for MBC determinations. Experiments were performed twice in triplicate.

### Time-Kill Kinetics Assays

Time-kill kinetics of selected DHP-based HHQ derivatives were conducted as previously described (González et al., 2019a). DHPs at concentrations of twice (2×) their MIC values were individually mixed with a freshly prepared suspension of *H. pylori* strain ATCC 700684 at ~10^6^ CFU/ml in BHI + FBS. DMSO instead of DHP was used as negative control in all assays. Mixtures of bacteria and DHPs were incubated under microaerobic conditions (85% N_2_, 10% CO_2_, and 5% O_2_) at 37°C with mild shaking. Aliquots of 10 μl were aseptically taken at time intervals of 0, 2, 4, 8, and 24 h after exposure, and seeded on Blood Agar Base No. 2 supplemented with 8% defibrinated horse blood. Plates were incubated at 37°C for 72 h under microaerobic conditions and CFU were determined. Experiments were performed twice in triplicate, and the results were presented as log10 CFU/ml versus incubation time. Statistical significances were considered if *p* < 0.05, according to the Mann–Whitney U test.

### Checkerboard Assays

Potential antimicrobial synergies between selected DHP-based HHQ derivatives and conventional antibiotics including metronidazole, clarithromycin and levofloxacin were evaluated by the checkerboard assay (González et al., 2019b). As a first step, the pairs of compounds to be evaluated were twofold serially diluted in BHI + FBS using two microtiter plates; one compound was diluted along all the rows of a first plate, while the other compound was diluted along all the columns of a second plate. Next, equal volumes of both gradients were mixed in a third microtiter plate, which were immediately inoculated with a freshly prepared bacterial suspension of *H. pylori* adjusted at 2 × 10^6^ CFU/ml in BHI + FBS. Plates were incubated at 37°C for 48 h under microaerobic conditions, and then, the microbial growth was colorimetrically revealed by the addition of 0.01 mg/ml resazurin. Fractional inhibitory concentration index (FICI) was calculated as FIC_A_ (MIC_A_ in the presence of B/MIC_A_ alone) + FIC_B_ (MIC_B_ in the presence of A/MIC_B_ alone), where A and B represent two different antimicrobial agents. Values of FICI ≤ 0.5, 0.5 < FICI ≤ 1, 1 < FICI ≤ 4, and FICI > 4 indicate synergistic, additive, neutral or antagonist interactions, respectively (González et al., 2019a,b).

### HsrA Expression and Purification

The HsrA response regulator from *H. pylori* strain 26695 (ATCC 700392) was overexpressed in *E. coli* BL21(DE3), and purified by immobilized metal-affinity chromatography (IMAC) according to previously described procedures (González et al., 2019b). The recombinant protein was finally dialyzed against the store buffer [50 mM Tris–HCl (pH 8), 300 mM NaCl, 10% glycerol] and conserved at −20°C until use. Protein concentration was determined using the BCA™ Protein Assay kit (Thermo Fisher Scientific, Bothell, WA, United States).

### Electrophoretic Mobility Shift Assays

The inhibitory action of DHP-based HHQ derivatives on the *in vitro* DNA binding activity of HsrA was assessed by electrophoretic mobility shift assay (EMSA), as previously described (González et al., 2019b). Briefly, recombinant HsrA protein (5 μM) was mixed with 120 ng of its target promoter (P*porGDAB*) in a 20 μl reaction volume containing 10 mM bis-Tris (pH 7.5), 40 mM KCl, 100 mg/L BSA, 1 mM DTT and 5% glycerol, in the presence of 3, 2, and 1 mM of each 1,4-DHP derivative. DMSO instead of DHP was included as vehicle control, while an internal sequence of the gene *pkn22* (*alr2502*) from *Anabaena* sp. PCC 7120 was used as non-specific competitor DNA in all assays. Mixtures were incubated at room temperature for 20 min and subsequently separated in a 6% native polyacrylamide gel electrophoresis. Gels were stained with SYBR Safe® (Thermo Fisher Scientific) and analyzed by using a Bio-Rad Gel Doc 2000 Imaging System (Bio-Rad Laboratories, Hercules, CA, United States).

### Isothermal Titration Calorimetry Assays

Isothermal titration calorimetry (ITC) experiments were carried out in an Auto-iTC200 calorimeter (MicroCal, Malvern-Panalytical, Malvern, United Kingdom) in order to determine several thermodynamic parameters of the molecular interaction between HsrA and selected DHP derivatives. The protein, located in the calorimetric cell at 20 μM, was titrated with each ligand, located in the injection syringe at 200 μM, by programming a series of 19 injections of 2 μl, with 150 s time spacing, 10 μcal/s reference power, and 750 rpm stirring speed (Velázquez-Campoy et al., 2015). Experiments were performed in buffer 50 mM Tris–HCl (pH 8), 150 mM NaCl, 10% glycerol, 1% DMSO at two different temperatures (15°C and 25°C) in order to obtain the best signal. The heat effect per injection was normalized by the amount of ligand injected, and the interaction isotherm was analyzed by nonlinear least-squares regression data analysis considering an interaction model with a single ligand binding site in the protein, using user-defined fitting routines in Origin 7.0 (OriginLab, Northampton, MA, United States).

### Molecular Docking Analysis

The tridimensional structure of the *H. pylori* HsrA response regulator (2HQR, model 1, chain A) was retrieved from the Protein Data Bank.^1^ The 3D structures of selected DHPs were built in Corina Classic^2^ and energy minimized using the AMMOS software (Pencheva et al., 2008). If compounds contain an unspecified stereocenter, both enantiomers were built for each ligand and used in docking studies. The protein and the ligands were prepared using the AutoDockTools 1.5.6 program. Molecular docking analyses were performed using AutoDock Vina (Trott and Olson, 2010). Rotatable bonds were defined as free for the ligands and rigid for the protein. The AutoGrid4 algorithm was used to estimate the interaction energy of each ligand pose. The pose that exhibited the lowest free energy of interaction (Δ*G*) for each ligand was considered as its predicted model of binding to the target protein. HsrA-ligand complex structures were visualized by PyMOL.^3^

### *In vivo* Inhibition of HsrA and RNA Isolation

*Helicobacter pylori* strain 26695 (ATCC 700392) was grown during 48 h at 37°C on Blood Agar Base No. 2 supplemented with 8% defibrinated horse blood under microaerobic conditions (85% N_2_, 10% CO_2_, and 5% O_2_). Cells from four blood agar plates were aseptically resuspended in BHI + FBS and diluted to 80 ml at OD_600_ = 0.1 (~10^7^ CFU/ml) in the same medium. BHI broth cultures were incubated at 37°C for 24 h under microaerobic conditions in T75 culture flasks. Next, cultures were pooled and subsequently divided into equal samples of 12 ml, which were exposed either to 4 × MIC (16 mg/L) of the DHP-based HHQ derivative MD7, or to the same volume of DMSO (vehicle). After 2 h of exposure under microaerobic conditions, 1.5 ml of ice-cold RNA stop solution (95% EtOH, 5% acid-buffered phenol) was added to each culture in order to preserve RNA integrity. Bacterial cells were immediately harvested by centrifugation (10,000 rpm for 5 min) at 4°C, washed once with ice-cold 50 mM Tris–HCl pH 7.4, 100 mM EDTA, and then lysed to extract total RNA as previously described (Sarasa-Buisan et al., 2021). Genomic DNA was removed with the TURBO DNA-free™ Kit (Thermo Fisher Scientific). The absence of residual DNA in RNA preparations was assessed by qPCR. Quality of RNA samples was checked using a NanoDrop spectrophotometer (Thermo Scientific) and agarose gel electrophoresis.

### Quantitative Real-Time PCR

Reverse transcription of 2 μg of total RNA from each sample was carried out using SuperScript retrotranscriptase (Invitrogen) in 40 μl of reaction volume containing 150 ng of random primers (Invitrogen), 1 mM dNTP mix (GE Healthcare) and 10 mM DTT. Real-time PCR (qPCR) was performed using the QuantStudio™ 5 Real-Time PCR System (Applied Biosystems). Each reaction was set up in a final volume of 30 μl containing 10 ng of cDNA template, 12.5 μl of SYBR Green PCR Master Mix and 160 nM of each primer. Negative controls with no cDNA were included. The sequences of primers used for transcript quantification of selected genes are defined in Supplementary Table S1. Relative quantification was performed according to the ΔΔCt method (Livak and Schmittgen, 2001). Expression levels were normalized using 16S rRNA as housekeeping gene.

### Cytotoxicity and Therapeutic Index

The *in vitro* toxicity of selected DHP-based HHQ derivatives toward HeLa cells was determined by the PrestoBlue™ assay (Thermo Fisher Scientific), according to the manufacturer’s instructions. Briefly, cells were cultured in Dulbecco’s modified Eagle’s medium containing 10% fetal bovine serum, 1% L-Glutamax solution and 1% penicillin/streptomycin solution using a humid incubator at 37°C with 5% CO_2_ until 80% confluence was achieved. Next, cells were detached with 0.25% trypsin, counted with a Neubauer chamber, and seeded in 96-well microplates at a density of 10,000 cells per well. Then, cells were allowed to adhere for 24 h and subsequently exposed to either DMSO (1.2% final concentration) or selected DHP derivatives at a range of concentrations from 128 to 0.125 mg/L. After 24 h of exposure, the PrestoBlue cell viability reagent was added to each well at 10% v/v and plates were incubated for another 2 h under the same conditions. The absorbance of each well was measured using a Synergy HT microplate reader (Excitation, 530 nm; Emission, 590 nm, BioTek Instruments, Winooski, United States). Experiments were performed twice in triplicate. The 50% cytotoxic concentration (CC_50_) was defined as the compound concentration required for the reduction of cell viability of DMSO (vehicle)-treated cell cultures by 50%, and it was calculated by regression analysis using Microsoft Excel. Therapeutic index (TI) values for each compound of interest were calculated as the ratio of the CC_50_ (cytotoxic activity) to the MIC (antibacterial activity) (Pitucha et al., 2016).

## Results

### DHP-Based HHQ Derivatives Exhibited Strong Bactericidal Activities Against Antibiotic-Resistant Strains of *Helicobacter pylori*

**Figure 1:**
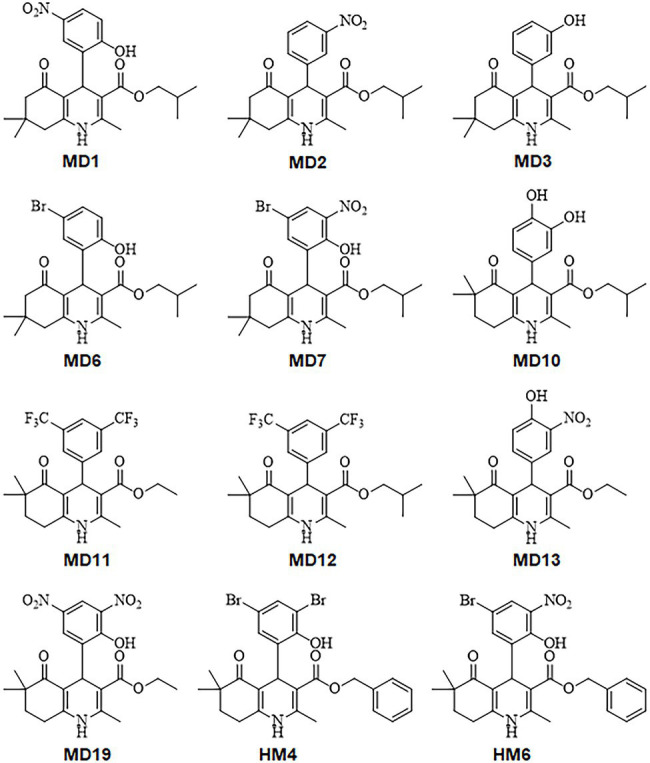
Chemical structures of the 1,4-dihydropyridine (DHP)-based hexahydroquinoline (HHQ) derivatives evaluated in this work.

Twelve DHP derivatives in which substituted cyclohexane rings were fused to the 1,4-DHP ring leading to a condensed ring system (HHQ) were analyzed regarding their potential as novel antimicrobial candidates against *H. pylori* (Figure 1). All the DHP-based HHQ derivatives evaluated in this work had previously shown significant detriments in their abilities to block L- and T-type calcium channels (Schaller et al., 2018; Aygün Cevher et al., 2019). Although all of these compounds carry an HHQ core, they differ from each other by their substitution patterns at the C-4 phenyl ring, the type of the alkyl group at the C-3 ester functionality, and the position of two additional methyl substituents at C6/C7 of the HHQ ring (Figure 1; Supplementary Table S2).

MIC and MBC values of all DHP derivatives were determined against three different antibiotic-resistant strains of *H. pylori*, showing resistance to clarithromycin (ATCC 700684), metronidazole (ATCC 43504), and levofloxacin (Donostia 2). As shown in Table 1, at least six condensed DHP derivatives (MD1, MD2, MD6, MD7, HM4, and HM6) exhibited strong bactericidal activities against all the *H. pylori* resistant-strains tested, with MIC values in the range of 1–8 mg/L. While compounds MD10 and MD19 exhibited moderate anti-*H. pylori* activities, other DHPs like MD3, MD12, and MD13 appeared poorly effective as antimicrobials. No relevant differences were observed in the antimicrobial activities of these molecules with respect to the antibiotic-resistance pattern of the *H. pylori* strains used in the assays.

**Table 1:** Minimal inhibitory and bactericidal concentrations of 12 DHP-based HHQ derivatives against different antibiotic-resistant strains of *H. pylori.*

DHP	MIC (MBC), mg/L		
	ATCC 700684 (CLR-R)	ATCC 43504 (MTZ-R)	Donostia 2 (LVX-R)
MD1	8 (8)	8 (8)	8 (8)
MD2	8 (8)	8 (8)	4 (8)
MD3	>64 (>64)	64 (64)	64 (64)
MD6	4 (4)	4 (4)	4 (4)
MD7	4 (4)	4 (8)	4 (8)
MD10	32 (64)	32 (32)	32 (64)
MD11	64 (64)	64 (64)	32 (64)
MD12	>64 (>64)	>64 (>64)	>64 (>64)
MD13	>64 (>64)	64 (>64)	>64 (>64)
MD19	32 (32)	64 (64)	32 (64)
HM4	2 (2)	2 (4)	4 (4)
HM6	1 (1)	2 (2)	2 (2)
Clarithromycin	16 (32)	<0.03 (<0.03)	<0.03 (<0.03)
Metronidazole	1 (2)	64 (128)	8 (8)
Levofloxacin	0.125 (0.125)	0.5 (0.5)	16 (32)

MIC, minimal inhibitory concentration; MBC, minimal bactericidal concentration; CLR-R, clarithromycin-resistant strain; MTZ-R, metronidazole-resistant strain; and LVX-R, levofloxacin-resistant strain.

**Figure 2:**
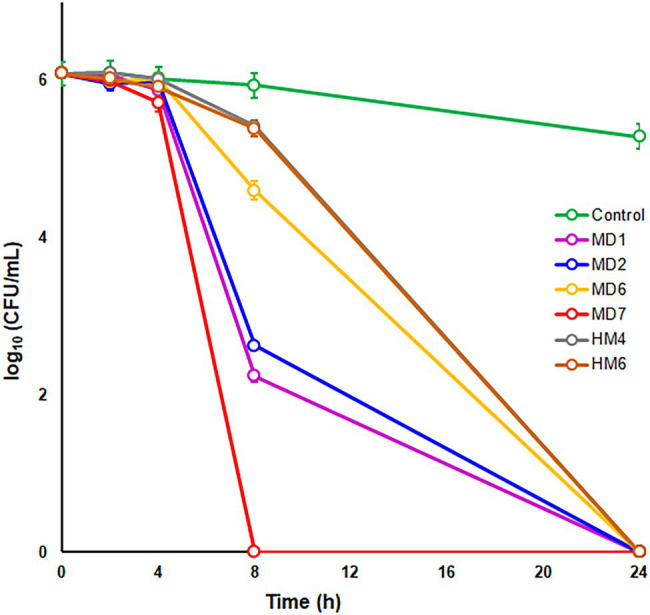
Time–kill kinetics of selected DHP-based HHQ derivatives against *Helicobacter pylori* strain ATCC 700684. Bacterial counts were determined at time zero and after 2, 4, 8, and 24 h of exposure to two times the MIC of each compound. Mixtures of bacteria with dimethyl sulfoxide (DMSO; vehicle) instead of DHP were used as controls. Values are the averages of six independent determinations; vertical bars represent SDs. Please note that in some instances, the error bar is smaller than the symbols used.

To further characterize the bactericidal activities of most effective DHP-based HHQ derivatives, time-kill kinetic assays were carried out by exposing the *H. pylori* strain ATCC 700684 to 2 × MIC of compounds MD1, MD2, MD6, MD7, HM4, and HM6 (Figure 2). Despite the fact that no CFUs could be detected after 24 h of exposure to this concentration of any of the DHP evaluated, significant differences were appreciated in the rate of killing produced by each compound from 8 h of exposure. Thus, the decline in bacterial counts occurred significantly faster (*p* < 0.05) after treatment with MD7, MD1, and MD2. Notably, MD7 was completely lethal at 8 h.

In order to preliminarily estimate undesirable side effects of these novel DHP-class antimicrobials, we determine the MIC/MBC values of MD1, MD2, MD6, MD7, HM4, and HM6 against both a Gram-negative and a Gram-positive representative species of the human normal microbiota. As shown in Table 2, none of the six most bactericidal DHPs against *H. pylori* exhibited relevant antimicrobial effects against *E. coli* or *S. epidermidis*, which could suggest a specific mechanism of action of these compounds against a molecular target expressed by *H. pylori* or *Epsilonproteobacteria*, not shared with other bacterial families.

**Table 2:** Antimicrobial activities of selected DHP-based HHQ derivatives against two representative species of the human normal microbiota.

DHP	MIC (MBC), mg/L	
	*E. coli* ATCC 25922	*S. epidermidis* ATCC 12228
MD1	>64 (>64)	>64 (>64)
MD2	>64 (>64)	>64 (>64)
MD6	>64 (>64)	>64 (>64)
MD7	>64 (>64)	>64 (>64)
HM4	>64 (>64)	>64 (>64)
HM6	>64 (>64)	>64 (>64)
Ampicillin	4 (4)	4 (4)

MIC, minimal inhibitory concentration and MBC, minimal bactericidal concentration.

The FICI values calculated after the exposure of *H. pylori* to MD1, MD2, MD6, MD7, HM4, and HM6 in combination with either clarithromycin, metronidazole or levofloxacin resulted in the range between >1 and ≤4 in all cases, according to checkerboard assays. Hence, no synergistic or additive effects appeared to occur with the use of these novel anti-*H. pylori* compounds in combination with conventional first-line antibiotics.

### Bactericidal DHP-Based HHQ Derivatives Inhibited the Biological Activity of the *Helicobacter pylori* Essential Response Regulator HsrA

**Figure 3:**
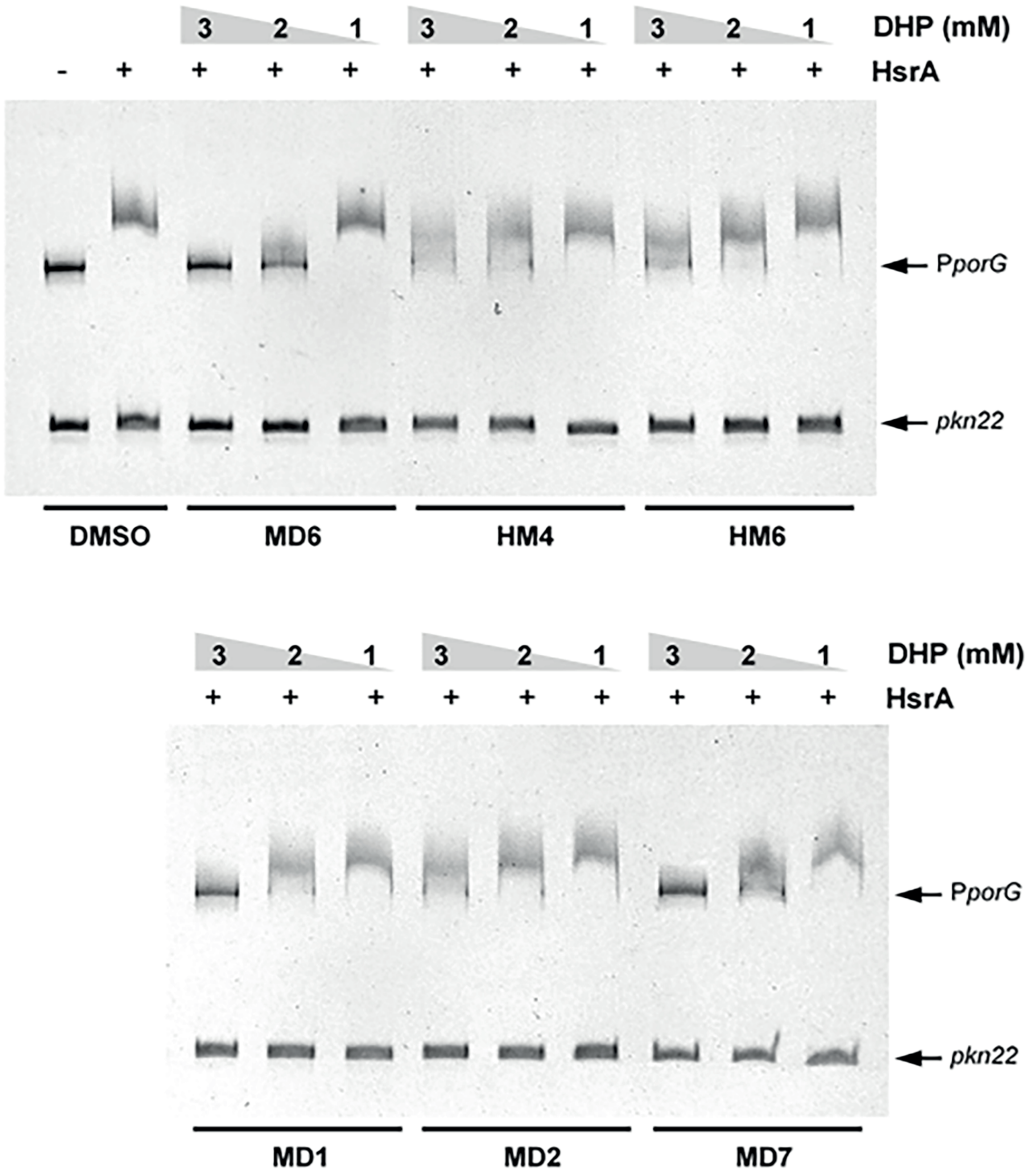
Electrophoretic mobility shift assays (EMSAs) showing the ability of selected DHP-based HHQ derivatives to specifically inhibit the *in vitro* DNA binding activity of the *H. pylori* response regulator HsrA. The recombinant protein (5 μM) was mixed with 120 ng of target promoter (*porGDAB* operon) in the presence of 3, 2, and 1 mM of DHP. The *Anabaena* gene *pkn22* was included as non-specific competitor DNA in all assays. Mixture of protein and DNA with DMSO (vehicle) instead of DHP was used as control. Protein-DNA interactions were analyzed by 6% PAGE using SYBR Safe® staining. Representative gel images are shown in a black-white inverted mode.

Previous studies have demonstrated that several commercially available DHP-class antihypertensive drugs act as low-molecular-weight ligands of the *H. pylori* essential response regulator HsrA and sensibly inhibited the *in vitro* DNA binding activity of this protein (González et al., 2019a). In order to evaluate *in vitro* the inhibitory action of the most effective anti-*H. pylori* DHP-based HHQ derivatives, we carried out EMSA experiments in the presence of increasing concentrations of MD1, MD2, MD6, MD7, HM4, and HM6 (Figure 3).

All the DHPs tested affected the *in vitro* affinity of HsrA by its target promoter P*porGDAB*. However, due to the poor solubility of DHP-based HHQ derivatives in aqueous solutions like the EMSA reaction buffer, the differences observed in the magnitude of binding inhibition could not be strictly associated with corresponding differences in the affinity of such ligands by HsrA. This thermodynamic parameter of DHP-HsrA interactions was subsequently evaluated by ITC.

In addition to the *in vitro* EMSA experiments, the inhibitory action of DHPs on the regulatory activity of HsrA *in vivo* was assessed by quantitative real-time PCR (qPCR). For this purpose, a cell suspension of *H. pylori* strain 26695 at 10^7^ CFU per ml in BHI broth +4% FBS was exposed during 2 h to 4 × MIC (16 mg/L) of the DHP-based HHQ derivative MD7. At this time, cells were treated with RNA stop solution (95% EtOH, 5% acid-buffered phenol) and total RNA was extracted. qPCR analyses were carried out in order to evaluate changes in the transcript abundance of genes *porA* (*hp1110*) and *tlpB* (*hp0103*), which have been previously recognized as targets of HsrA transcriptional activation (Olekhnovich et al., 2014). The 16S rRNA gene (*hprrnA16S*) was used as housekeeping gene, while *nixA* (*hp1077*) was included as negative control (Pelliciari et al., 2017).

**Figure 4:**
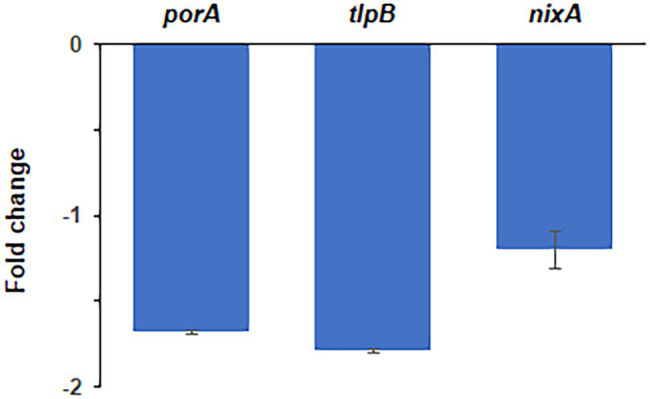
Quantitative real-time PCR (qPCR) analysis of transcript abundance changes of selected genes in response to the exposure to lethal concentrations of the DHP-based HHQ derivative MD7. Total RNA was extracted from *H. pylori* strain 26695 after 2 h exposure to 4 × MIC (16 mg/L) of MD7. Relative transcription of genes *porA*, *tlpB* and *nixA* in MD7-treated cells with respect to DMSO (vehicle) treated cells are indicated as fold changes. Values correspond to the average of two independent biological samples, each analyzed in three technical replicates. Error bars indicate the SDs.

Exposure of *H. pylori* cells to lethal concentrations of the HsrA inhibitor MD7 during 2 h induced a 1.7-fold decrease in the abundance of *porA* transcripts and 1.8-fold decrease in the level of *tlpB* mRNA with respect to DMSO (vehicle)-treated cells. However, the treatment with this HsrA inhibitor did not lead to an appreciable change in the transcript level of *nixA*, a gene without a direct control of HsrA (Figure 4).

### DHP-Based HHQ Derivatives Interact With the DNA-Binding Domain of HsrA at 1:1 Stoichiometry in the Micromolar Range

Thermodynamic parameters of the molecular interactions between HsrA and its bactericidal inhibitors MD1, MD2, MD6, MD7, HM4, and HM6 were analyzed by isothermal titration calorimetry (ITC). As previously observed with other HsrA inhibitors, DHP-based HHQ derivatives interacted with this response regulator following a 1:1 stoichiometry, that is, each HsrA monomer binds one molecule of DHP (Supplementary Figure S1). Despite all HsrA-DHP complexes showed dissociation constants in the micromolar range, some little differences in the binding affinities of these ligands could be intuited according to their *K*_d_ values, which appear to suggest that MD1, HM4, and HM6 interact with more affinity with the protein than the rest of the ligands tested (Table 3).

**Table 3:** Thermodynamic parameters and interacting amino acid residues of the protein-ligand complexes formed between HsrA and selected DHP-based HHQ derivatives, according to ITC and molecular docking analyses.

DHP	ITC^1^			Molecular docking^2^
	*K*_d_ (**μ**M)	Δ*H* (kcal/mol)	Δ*G* (kcal/mol)	Interacting residues
MD1	3.5	−1.5	−7.4	I135, V144, F149, L152, **K194**, **M195**, **P198**, **L199**
MD2	16	−3.1	−6.5	I135, Y137, V144, K145, G146, **K194**, **P198**
MD6	25	−2.0	−6.3	I135, Y137, V142, V144, P148, F149, L152, **K194**, **P198**, **L199**
MD7	23	−7.8	−6.3	I135, Y137, V142, V144, F149, L152, **K194**, **M195**, **P198**, **L199**
HM4	4.0	−0.7	−7.4	I135, Y137, V142, V144, G146, P148, F149, **K194**, **P198**
HM6	5.4	−2.7	−7.2	I135, Y137, V142, V144, G146, P148, **K194**, **P198**

1 Relative error in *K*_d_ is 15%, absolute error in Δ*H* is 0.4 kcal/mol, absolute error in Δ*G* is 0.1 kcal/mol.

2 Amino acid residues directly involved in forming the helix-turn-helix (HTH) DNA binding motif of HsrA are highlighted in bold fonts.

**Figure 5:**
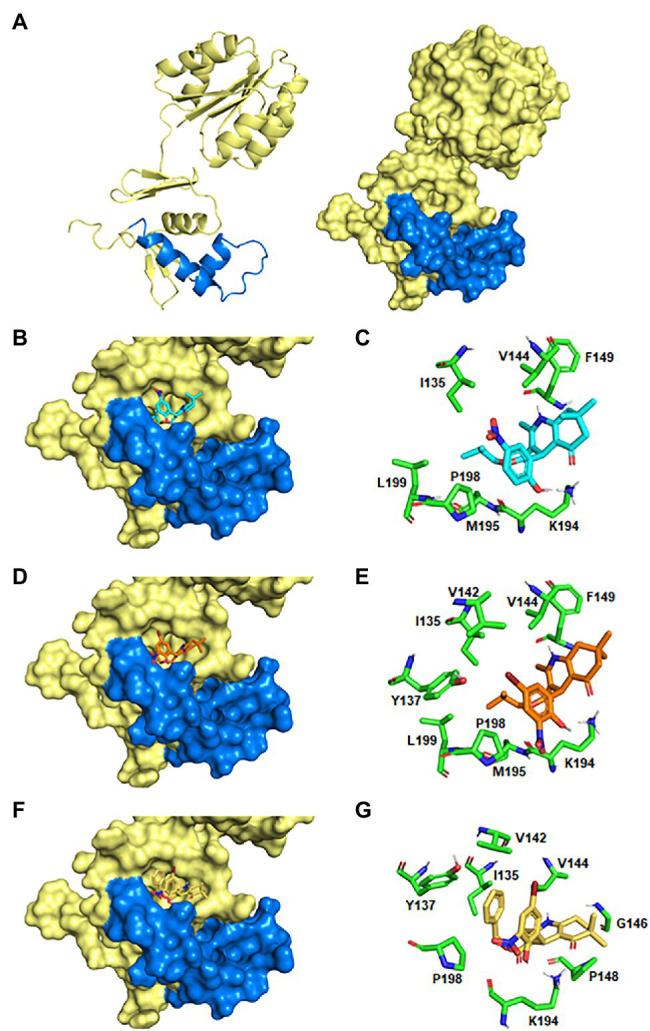
Predicted models of HsrA-DHP interactions. **(A)** Ribbon diagram and molecular surface of an HsrA monomer. The helix-turn-helix (HTH) DNA binding motif in the C-terminal domain has been highlighted in blue. **(B,D,F)** 3D views of the best-ranked docking poses of MD1, MD7, and HM6. **(C,E,G)** Detailed views of the HsrA amino acid residues involved in the interaction with the respective DHP.

Notably, the molecular docking analyses predicted that all the six highly bactericidal DHP-based HHQ derivatives interact with HsrA in the same binding site, consisting in a pocket on the surface of the C-terminal DNA binding domain which involved several amino acid residues of the helix-turn-helix (HTH) DNA binding motif (Table 3; Figure 5). This ligand-binding pocked is predominantly shaped by nonpolar residues including I135, V142, V144, G146, P148, F149, L152, M195, P198, and L199, but also comprises few polar amino acids such as Y137, K145, and K194.

Analysis of the best-ranked pose of each DHP ligand inside this common binding pocket revealed similar interaction patterns, with slight changes in the spatial arrangement of the DHP molecule promoted by differences in the chemical properties, sizes and positions of the substituent groups on the DHP scaffold. Several non-covalent interactions between these DHP ligands and neighboring HsrA amino acids appeared to define little differences in the affinity of each inhibitor by their target regulator. While some intermolecular interactions appear inherent to the DHP-based HHQ structure, other contacts are established or reinforced with dependence on the type of substituent. Thus, the phenyl ring present in all DHPs establishes a CH/π interaction with P198, while additional hydrophobic interactions occur between the HHQ condensed ring system and V144. In addition, the carbonyl group of the condensed ring interacts by hydrogen bonds with the NH_3_^+^ group of the K194 side chain (Figure 5).

The hydroxyl substituent in the *ortho* position of the phenyl ring in MD1, MD6, MD7, HM4, and HM7 forms a hydrogen bond with the NH_3_^+^ group of the K194 side chain, but this interaction is absent in MD2. The bromine substituent in the *meta* position of the phenyl ring in MD6, MD7, HM4, and HM6 could perform halogen bonding with the hydroxyl oxygen of Y137 side as well as hydrophobic interactions with I135, V142 and V144 (Figures 5E,G); however, the nitro group at this position does not appear to form any favorable interaction with the protein. Similarly, the dimethyl group at the condensed ring system in MD1, MD2, MD6, and MD7 is not involved in any favorable interactions, but the change in the position of this substituent in HM4 and HM6 allowed additional hydrophobic contacts with G146 and P148 (Figure 5G). The isobutyl ester moiety of MD1, MD2, MD6, and MD7 establishes hydrophobic interactions with adjacent I135, V142, Y137, L199; however, the conformation of ligand-protein complexes could be further stabilized in HM4 and HM6 by π-π stacking interactions of their benzyl ester moiety with Y137 and CH/π interactions with P198 (Figure 5G).

### Most of the Highly Bactericidal DHP-Based HHQ Derivatives Exhibited Cytotoxicity Levels Comparable With Those of Commercial DHP Drugs in HeLa Cells

Due to the absence of previous reports related to the cytotoxic potential of DHP-based HHQ derivatives, cytotoxicity studies were conducted in HeLa cells by the method of PrestoBlue (Lall et al., 2013). Cell cultures were exposed during 24 h to selected DHP-based HHQ derivatives at concentrations ranging from the lowest MIC value previously determined for *H. pylori* (1 mg/L) to more than 120 times this value. Three commercially available DHP-class antihypertensive drugs (nicardipine, nimodipine, and lercanidipine) were included as control in the assays.

**Figure 6:**
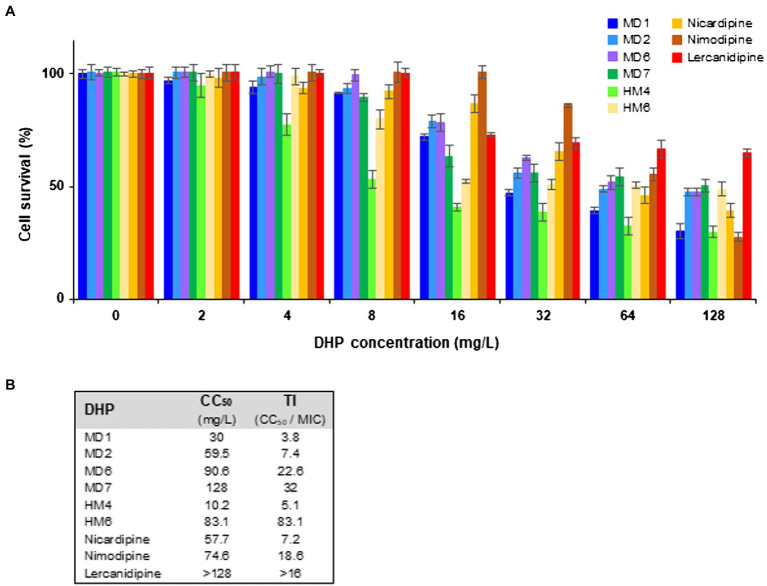
Cytotoxicity and therapeutic index of several DHP-class anti-*H. pylori* compounds. **(A)** Cytotoxicity of selected DHP-based HHQ derivatives and some commercial DHP drugs toward HeLa cells was assessed at 24 h of exposure through the PrestoBlue method. Experiments were performed twice in triplicate, vertical bars represent SDs. **(B)** The 50% cytotoxic concentration (CC_50_) was defined as the compound concentration that reduced the viability of DMSO (vehicle)-treated cell cultures by 50%. The indicated therapeutic index (TI) values were calculated as the ratio between CC_50_ and the MIC value for the *H. pylori* strain ATCC 700684.

As shown in Figure 6A, no relevant cytotoxicity was observed at 4 mg/L with most of the DHP derivatives tested, with the exception of HM4, which reduced HeLa cell viability to 77% at this concentration. The 50% cytotoxic concentration (CC_50_) of MD2, MD6, MD7, and HM6 was comparable to those exhibited by commercial DHP drugs (Figure 6B). However, the therapeutic index (TI) values, which were calculated as the ratio between CC_50_ and MIC, resulted higher than 3 in all cases. This fact supposes a wide therapeutic window for all of these DHP derivatives as anti-*H. pylori* antimicrobial candidates.

## Discussion

Antimicrobial resistance is nowadays a major global challenge for public health. The constant development of new resistance mechanisms, the unstoppable spread of antibiotic resistance genes among veterinary and clinical relevant pathogens but also in commensal bacteria, and the rapid dissemination of multidrug-resistant strains in a globalized world already threaten our capacities to face some infectious diseases (Chen et al., 2017; Ofori-Asenso, 2017; Nkansa-Gyamfi et al., 2019; Karakonstantis et al., 2020). In recent years, the efficacies of several antimicrobial combinatory therapies commonly used to eradicate infection by the carcinogenic bacterium *H. pylori* have drastically decreased worldwide because of an increasing emergence of resistance to first-line antibiotics (Boyanova et al., 2019; Kuo et al., 2021). Efforts are underway to discover new antimicrobial candidates directed against novel therapeutic targets in this pathogen that allow for overcoming the current resistome (Krzyzek et al., 2019, 2020; González et al., 2019b, 2020; Roszczenko-Jasinska et al., 2020; Salillas and Sancho, 2020).

In a previous work, we found that several antihypertensive drugs of the DHP class targeted the *H. pylori* response regulator HsrA and inhibited its essential function *in vitro*. Such DHP derivatives exhibited strong bactericidal activities against antibiotic-resistant strains of *H. pylori* and significantly reduced gastric colonization by this pathogen in the mouse model (González et al., 2019a); however, the hypotensive activity of these highly prescribed drugs could slow down their repurposing as novel antimicrobials.

1,4-DHP is one of the most privileged heterocyclic scaffolds in medicinal chemistry covering a broad spectrum of biological activities and therapeutic effects including antihypertensive, antianginal, neuroprotective, antioxidant, anti-inflammatory, anticancer, and antimicrobial (Ioan et al., 2011; Carosati et al., 2012; Mishra et al., 2019; Ling et al., 2021). Most 1,4-DHP derivatives share some structural features, such as the unsaturated 1,4-DHP core ring with unsubstituted N1 atom, small alkyl groups (usually methyl) at the C2 and C6 positions, ester groups at the C3 and C5 positions, and a phenyl ring with different substituent types and patterns at the C4 position (Olejnikova et al., 2014; Ling et al., 2021).

To further evaluate the potential of 1,4-DHP as a scaffold for novel antimicrobial drugs against *H. pylori*, we determined the antibacterial effect of 12 DHP-based hexahydroquinoline derivatives which have previously shown no significant blocking effects on calcium channels. As previously described with other biological activities exerted by this class of compounds, the observed antimicrobial effects against *H. pylori* of these DHP-based HHQ derivatives depended on the substitution pattern of the DHP scaffold. Thus, the most potent bactericidal activities against *H. pylori* were observed in the presence of the DHP derivatives HM4 and HM6. These two compounds are distinguished by possessing a benzyl moiety in the ester functionality at the C3 position, and dimethyl substituents at the C6 position in the HHQ condensed system, one or two bromine in the *meta* positions of the phenyl ring, and a hydroxyl group in the *ortho* position.

Notably, the change of the alkyl group of the ester moiety from benzyl (HM6) to isobutyl (MD7), and the modification of the position of the dimethyl group from C6 (HM6) to C7 (MD7) on the HHQ ring system, resulted in up to 4-fold reduction in the antimicrobial activity against *H. pylori*. These structure-associated differences in the anti-*H. pylori* activities between HM6 and MD7 were partially supported by differences in the binding affinities of these DHPs for their target HsrA, according to ITC and molecular docking analyses. However, MD7 led to a significantly faster decline in bacterial counts when compared to HM6 in time-kill kinetic assays, maybe associated to a faster translocation across the cell membrane because of its smaller molecular size. Reduction of the hydrophobicity of the ester moiety by changing the alkyl group from benzyl or isobutyl to ethyl led to a severe detriment of the antimicrobial potential of these DHPs against *H. pylori*.

The addition of one or two bromine atoms in the *meta* position of the phenyl ring favored the antimicrobial activity as the result of additional noncovalent interactions between the DHPs and the target HsrA protein. This effect was less evident with the nitro group at the same positions, while the inclusion of trifluoromethyl substituents appreciably reduced the anti-*H. pylori* activity. Likewise, the presence of only hydroxyl substituents in the phenyl ring resulted in a low antimicrobial potential.

The use of 1,4-DHP as a scaffold for novel antimicrobials has been the focus of several investigations since decades ago. Fiszer-Maliszewska and co-workers in 1985 observed that some DHPs inhibited the *in vitro* growth of antibiotic-resistant *Mycobacterium tuberculosis* strains at 3.1 mg/L (Fiszer-Maliszewska et al., 1985). Since then, the antitubercular properties of other DHP derivatives have been reported by many other researchers (Trivedi et al., 2011; Desai et al., 2015; Zandhaghighi et al., 2017; Venugopala et al., 2021). DHPs have been also evaluated as antimicrobials against other pathogenic bacteria including *S. aureus* (Ceviz et al., 1997; Olejnikova et al., 2014; Mahmoodi et al., 2015; Nkosi et al., 2016; Nosrati et al., 2021), *E. coli* (Murthy et al., 2012; Olejnikova et al., 2014; Mahmoodi et al., 2015; Nkosi et al., 2016; Ahamed et al., 2018), *Pseudomonas aeruginosa* (Mahmoodi et al., 2015; Nkosi et al., 2016; Ahamed et al., 2018), *Vibrio cholerae* (Lavanya et al., 2021) and *Klebsiella pneumoniae* (Murthy et al., 2012); but also against parasites (Núñez-Vergara et al., 1997; Palit and Ali, 2008; Reimao et al., 2010; Pollo et al., 2017; Jeddi et al., 2021) and fungi (Chhillar et al., 2006; Jezikova et al., 2017; Ahamed et al., 2018). This class of molecules have been also studied as inhibitors of bacterial transmembrane efflux pumps, acting thereby as enhancers of the action of conventional antibiotics (Lentz et al., 2016, 2018, 2019).

Several DHP-class antihypertensive drugs including nifedipine, nicardipine, nisoldipine, nimodipine, nitrendipine, and lercanidipine have previously shown MIC values in the range of 4–32 mg/L against different strains of *H. pylori* (González et al., 2019a). In the present study, the chemical modifications carried out on the 1,4-DHP scaffold led to a noticeable increase in the anti-*H. pylori* activity, with MIC values ranging from 1 to 4 mg/L in the case of compounds HM4 and HM6. This strong antimicrobial effect against *H. pylori* is comparable with those achieved by some first-line conventional antibiotics like metronidazole, and up to 4-fold greater than those previously observed with commercial 1,4-DHP drugs.

The MIC values of those DHP-based HHQ derivatives that exhibited the most potent bactericidal effects against *H. pylori* were in all cases >3 times higher than the concentration necessary to induce damage in HeLa cells (TI > 3), suggesting wide therapeutic windows of these molecules as potential new antimicrobial drugs. On the other hand, these novel anti-*H. pylori* compounds resulted poorly deleterious for *E. coli* and *S. epidermidis*, a Gram-positive and Gram-negative representative species of the human normal microbiota. These results might indicate a narrow-spectrum in the antimicrobial action of these novel compounds, and consequently, a reduced risk of associated dysbiosis (Francino, 2015; Konstantinidis et al., 2020). Our findings suggest that the mechanistic base of such specific antimicrobial action is based on the inhibitory effect of DHPs on the essential transcriptional regulatory activity of HsrA, an orphan response regulator unique in *Epsilonproteobacteria* (Muller et al., 2007; Olekhnovich et al., 2013; Pelliciari et al., 2017). Both *in vitro* and *in vivo* experiments showed an inhibition of the DNA binding activity of this regulatory protein in the presence of selected bactericidal DHPs, while ITC studies revealed HsrA-DHP interactions with 1:1 stoichiometry and dissociation constants in the micromolar range. These thermodynamic parameters of the molecular interactions between HsrA and the novel DHP derivatives are similar to those exhibited by all the low-molecular-weight inhibitors described so far for this protein (González et al., 2019a,b).

Notably, despite differences in the chemical structure, molecular size and physicochemical properties of six selected highly bactericidal DHP-based HHQ derivatives, all of these molecules appear to interact with HsrA in a common binding site located in a pocket on the protein surface at the C-terminal DNA binding domain (Hong et al., 2007), according to molecular docking predictions. This binding site, partially shaped by several amino acid residues directly involved in the HTH DNA binding motif, seemed to be occupied also by other previously recognized HsrA inhibitors including the natural flavonoids apigenin and hesperetin (González et al., 2019b), and the DHP-class antihypertensive drug nimodipine (González et al., 2019a). Hence, this predicted binding pocket could be a potential druggable binding site on HsrA, a validated therapeutic target in *H. pylori*.

Overall, the results presented here strongly support the use of 1,4-DHP as a scaffold for novel antimicrobials against *H. pylori*. New highly bactericidal 1,4-DHP derivatives showing MIC values against *H. pylori* comparable with those achieved by first-line antibiotics have been obtained. Molecular docking analysis of several HsrA-DHP interactions predicted a potential druggable binding pocket in the C-terminal DNA binding domain of this essential regulatory protein. Further studies should be carried out to experimentally validate and best characterize this binding pocket, which could open the door to structure-based design of improved HsrA inhibitors and lead to the definition of new strategies for drug discovery against *H. pylori* infection.

## Data Availability Statement

The original contributions presented in the study are included in the article/Supplementary Material; further inquiries can be directed to the corresponding author.

## Author Contributions

AG and MG designed the study and wrote the manuscript. AG, JC, MG, BS, AV-C, CS-B, and MM performed the experiments. AG, JC, MG, AV-C, CS-B, and MM analyzed and validated the data. MF, EP, and ÁL gave important technical advices and supervised some experiments. MG, MF, and ÁL contributed to the funding acquisition. All authors have checked, read, and approved the final version of the manuscript.

## Funding

This research has been funded by the Government of Aragon (grants B25_17R, B25_20R, and E35_20R) and the Spanish Ministry of Economy, Industry and Competitiveness (grant PID2019-104889GB-I00).

## Conflict of Interest

The authors declare that the research was conducted in the absence of any commercial or financial relationships that could be construed as a potential conflict of interest.

## Publisher’s Note

All claims expressed in this article are solely those of the authors and do not necessarily represent those of their affiliated organizations, or those of the publisher, the editors and the reviewers. Any product that may be evaluated in this article, or claim that may be made by its manufacturer, is not guaranteed or endorsed by the publisher.

## Footnotes

^1^ https://www.rcsb.org/

^2^ https://mn-am.com/

^3^ https://pymol.org/
