# Supplementary material for: 1,4-Dihydropyridine as a Promising Scaffold for Novel Antimicrobials Against Helicobacter pylori
Source: Front Microbiol. 2022 May 25;13:874709. doi: 10.3389/fmicb.2022.874709 (PMC9174938; doi:10.3389/fmicb.2022.874709)
Supplement: Supplementary file 1 [file Table_1.DOCX]

1,4-Dihydropyridine as a Promising Scaffold for Novel Antimicrobials against *Helicobacter pylori*

Andrés González, Javier Casado, Miyase Gözde Gündüz, Brisa Santos, Adrián Velázquez-Campoy, Cristina Sarasa-Buisan, María F. Fillat, Milagrosa Montes, Elena Piazuelo, Ángel Lanas

**Table S1.** List of oligonucleotides used in qPCR experiments.

| **Oligo ID** | **Sequence 5´- 3´** |
| --- | --- |
| qPCR-16SRNAup | CTGAGAGGGTGAACGGACACACTG |
| qPCR-16SRNAdw | CGTTGCTGCTTCAGGGTTTCCC |
| qPCR-PorAup | GGTGCGCGTGCCTACTATTGTC |
| qPCR-PorAdw | CGCCCACGAATTGGTAAGCC |
| qPCR-TlpBup | GAGCATGAAAGATTCCTCAACCACC |
| qPCR-TlpBdw | CGTTTCAATCAAACGCTTCCTTAACC |
| qPCR-NixAup | GCGCGATTAAAGTGAGTATGGTGGG |
| qPCR-NixAdw | CCCAGTCATACGCCTTGAGCATG |

| 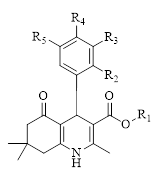 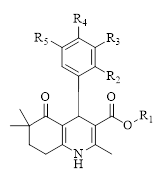  MD1-3, MD6, MD7 MD10-13, MD19, HM4, HM6 | | | | | | | |
| --- | --- | --- | --- | --- | --- | --- | --- |
| **DHP** | **R_1_** | **R_2_** | **R_3_** | **R_4_** | **R_5_** | **Molecular Weight** | **cLogP** |
| MD1 | CH_2_CH(CH_3_)_2_ | OH | H | H | NO_2_ | 428 | 4.44 |
| MD2 | CH_2_CH(CH_3_)_2_ | H | NO_2_ | H | H | 412 | 4.67 |
| MD3 | CH_2_CH(CH_3_)_2_ | H | OH | H | H | 383 | 4.26 |
| MD6 | CH_2_CH(CH_3_)_2_ | OH | H | H | Br | 462 | 5.22 |
| MD7 | CH_2_CH(CH_3_)_2_ | OH | NO_2_ | H | Br | 507 | 5.41 |
| MD10 | CH_2_CH(CH_3_)_2_ | H | OH | OH | H | 399 | 3.67 |
| MD11 | C_2_H_5_ | H | CF_3_ | H | CF_3_ | 475 | 5.77 |
| MD12 | CH_2_CH(CH_3_)_2_ | H | CF_3_ | H | CF_3_ | 503 | 6.70 |
| MD13 | C_2_H_5_ | H | NO_2_ | OH | H | 400 | 3.71 |
| MD19 | C_2_H_5_ | OH | NO_2_ | H | NO_2_ | 445 | 3.48 |
| HM4 | CH_2_C_6_H_5_ | OH | Br | H | Br | 575 | 6.21 |
| HM6 | CH_2_C_6_H_5_ | OH | NO_2_ | H | Br | 541 | 5.71 |

**Table S2.** Substitution patterns and some physicochemical properties of the DHPs tested in this work.

Physicochemical properties were calculated from the chemical structures using ChemDraw 20.0.


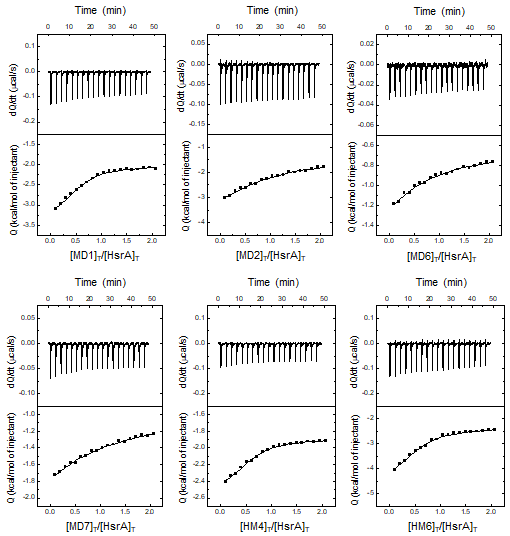


**Figure S1.** Isothermal titration calorimetry (ITC) analyses of the interaction between the *H. pylori* response regulator HsrA and the DHP derivatives MD1, MD2, MD6, MD7, HM4 and HM6. Upper panels show the thermograms (thermal power as a function of time), while lower panels show the binding isotherms (titrant normalised heat effects as a function of the ligand:protein molar ratio in the cell).
